# Elastic Asymmetry of PLA Material in FDM-Printed Parts: Considerations Concerning Experimental Characterisation for Use in Numerical Simulations

**DOI:** 10.3390/ma13010015

**Published:** 2019-12-18

**Authors:** Ma-Magdalena Pastor-Artigues, Francesc Roure-Fernández, Xavier Ayneto-Gubert, Jordi Bonada-Bo, Elsa Pérez-Guindal, Irene Buj-Corral

**Affiliations:** 1 Department of Strength of Materials and Structural Engineering (RMEE), Barcelona School of Industrial Engineering (ETSEIB), Universitat Politècnica de Catalunya–Barcelona Tech (UPC), 08028 Barcelona, Spain; francesc.roure@upc.edu (F.R.-F.); javier.ayneto@upc.edu (X.A.-G.); jordi.bonada@upc.edu (J.B.-B.); 2 Department of Strength of Materials and Structural Engineering (RMEE), Vilanova i la Geltrú School of Engineering (EPSEVG), Universitat Politècnica de Catalunya–Barcelona Tech (UPC), 08800 Vilanova i la Geltrú, Spain; elsa.perez@upc.edu; 3 Department of Mechanical Engineering (EM), Barcelona School of Industrial Engineering (ETSEIB), Universitat Politècnica de Catalunya–Barcelona Tech (UPC), 08028 Barcelona, Spain; irene.buj@upc.edu

**Keywords:** FDM, PLA, mechanical properties, bimodulus materials, standards, finite element analysis (FEA)

## Abstract

The objective of this research is to characterise the material poly lactic acid (PLA), printed by fused deposition modelling (FDM) technology, under three loading conditions—tension, compression and bending—in order to get data that will allow to simulate structural components. In the absence of specific standards for materials manufactured in FDM technology, characterisation is carried out based on ASTM International standards D638, D695 and D790, respectively. Samples manufactured with the same printing parameters have been built and tested; and the tensile, compressive and flexural properties have been determined. The influences of the cross-sectional shape and the specimen length on the strength and elastic modulus of compression are addressed. By analysing the mechanical properties obtained in this way, the conclusion is that they are different, are not coherent with each other, and do not reflect the bimodular nature (different behaviour of material in tension and compression) of this material. A finite element (FE) model is used to verify these differences, including geometric non-linearity, to realistically reproduce conditions during physical tests. The main conclusion is that the test methods currently used do not guarantee a coherent set of mechanical properties useful for numerical simulation, which highlights the need to define new characterisation methods better adapted to the behaviour of FDM-printed PLA.

## 1. Introduction

Additive manufacturing (AM) technologies allow for converting virtual models into physical models in a quick and easy way by means of tool-free processes. Different polymeric materials are being produced for 3D Printing (3DP) with a wider range of properties. 3DP has many applications in sectors such as automotive, electronics or medical [1]. Aliphatic polyesters, in particular poly lactic acid (PLA), are suitable materials for in vivo applications because of their biocompatibility, biodegradability, good mechanical strength and processability. PLA is the most researched and used aliphatic biodegradable polyester. It is a leading biomaterial for numerous applications in both medicine and industry, and the ability to adapt its properties for specific applications makes the market capacity of PLA products very broad, which has catalysed an extensive and growing amount of research aimed at the use of this material in innovative forms and applications [2,3,4,5,6,7].

If PLA-printed parts are to be usable as real industrial or biomechanical components, their structural and mechanical reliability has to be proved by means of strength and stiffness verifications. These will be done by classical strength of materials calculations or by finite element (FE) simulations. In either case, it is essential to have a coherent set of mechanical properties of the material under the different service conditions (tension, compression, bending, torsion, etc.). Flexural strength and tensile strength are two of the most commonly used values for comparing plastic materials. Compressive strength gives a good indication of short-term load capacities. Rigidity is expressed by the modulus of elasticity in tension and flexion. Reference data on the mechanical properties of PLA are available in the literature [2,8,9,10,11,12,13,14], with the tensile test being the most common of the tests performed to characterise the mechanical behaviour of PLA [15,16,17,18,19,20,21].

However, information of the bulk material behaviour is not always useful for carrying out numerical simulations to check the proper in-service behaviour of a fused deposition modelling (FDM)-printed component. To simulate the behaviour of a FDM-printed component, it is previously necessary to characterise the material in the same way that it is in the component. Many works start from the existing standards for polymeric materials to characterise also their FDM-printed versions. The ASTM standards D638 (tensile properties), D695 (compressive properties) and D790 (flexural properties) are widely used for this purpose.

The objective of this work was to obtain the mechanical properties of parts printed on PLA for use in numerical simulations, and to contrast the procedures for such purposes based on the use of ASTM standards. The results obtained considering an isotropic behaviour were compared for the different types of loads in the standards (tension, compression and bending). Nevertheless, when considering the bimodular behaviour of the material [22,23,24,25], inconsistencies were observed between the results obtained in the different tests. Differences in compression behaviour were also observed depending on the shape and proportions of the samples.

It is concluded that the currently accepted approach, based on characterisation according to the above standards (or the equivalent ISO standards), is not suitable for this purpose. It is necessary to define new characterisation methods that take into account of the bimodularity of the material and ensure a consistent set of mechanical characteristics for numerical simulation. This will be the next step of this research.

## 2. Test Methods

The PLA samples were 3D-printed with FDM technology, in a BCN3D Sigma v17 machine (BCN3D Technologies, Barcelona, Spain). It is an FDM desktop 3D printer with an independent dual extruder.

Specifications and properties of the PLA filament, provided by the manufacturer of the machine, are shown in Table 1.

The slicing software was Cura 0.1.5. All the specimens were manufactured with constant printing parameters, which are provided in Table 2.

Anisotropy as well as the influence of different printing parameters, like infill percentage or printing speed, were beyond the scope of this study.

ASTM (American Society for Testing and Materials) and ISO (International Organization for Standardization) mechanical testing standards are widely used to determine mechanical properties of plastics. These test procedures assume the material is continuous and homogeneous, although not necessarily isotropic. They do not include particular considerations for additive manufacturing [26,27]. Forster [28] reviews existing procedures for testing polymers and analyses their feasibility for additive manufacturing processes. It should be mentioned; however, that there are technical committees working on developing new standards for additive manufacturing (ISO/TC261, ASTM F42).

ASTM D638-02a Standard Test Method for Tensile Properties of Plastics [29], ASTM D695-02a Standard Test Method for Compressive Properties of Rigid Plastics [30] and ASTM D790-02 Flexural Properties of Unreinforced and Reinforced Plastics and Electrical Insulating Materials [31] were followed in the tests carried out.

As regards the shape and dimensions of the specimens tested, the recommendations of the standards were followed. For the tensile and bending tests, the options were quite clear; however, in the case of the compression test, two cross-section shapes were possible, and the specimen length depended on the mechanical property to be obtained.

### 2.1. Specimens

**Figure 1:**
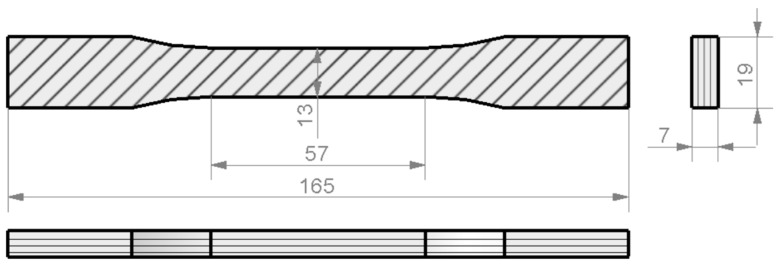
Drawing of the tensile test specimen (dimensions in mm).

**Figure 2:**
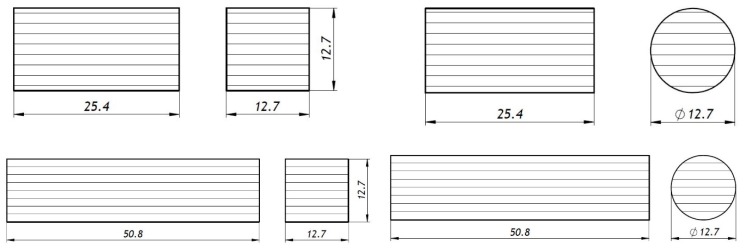
Drawing of the compression test specimens (dimensions in mm).

**Figure 3:**
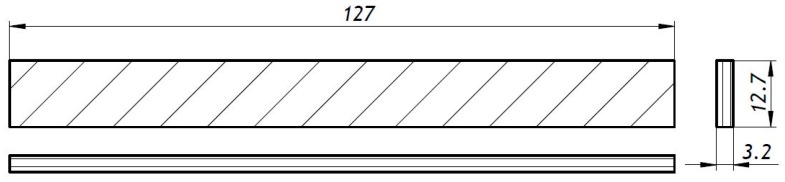
Drawing of the three-point bending test specimen (dimensions in mm).

The shape and dimensions of the specimens were defined in accordance with the standards (Figure 1, Figure 2 and Figure 3) as above mentioned, and six samples of each series were manufactured: One set of specimens for tensile tests, another set for bending tests, and four sets for compression tests, where two different cross-section shapes and two different specimen lengths were possible.

**Figure 4:**
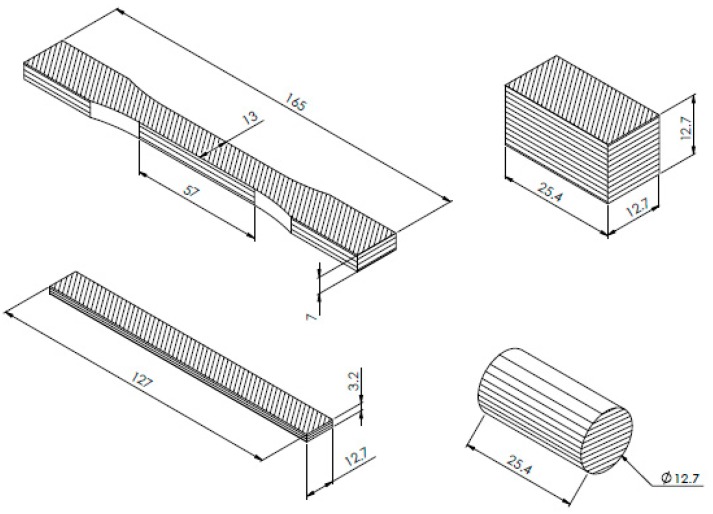
Structure of specimens with the raster and layers.

As mentioned above, the printing parameters were kept constant (Table 2). The layers were oriented in the direction of the stresses. Figure 4 shows the print raster and the direction of the layers.

**Figure 5:**
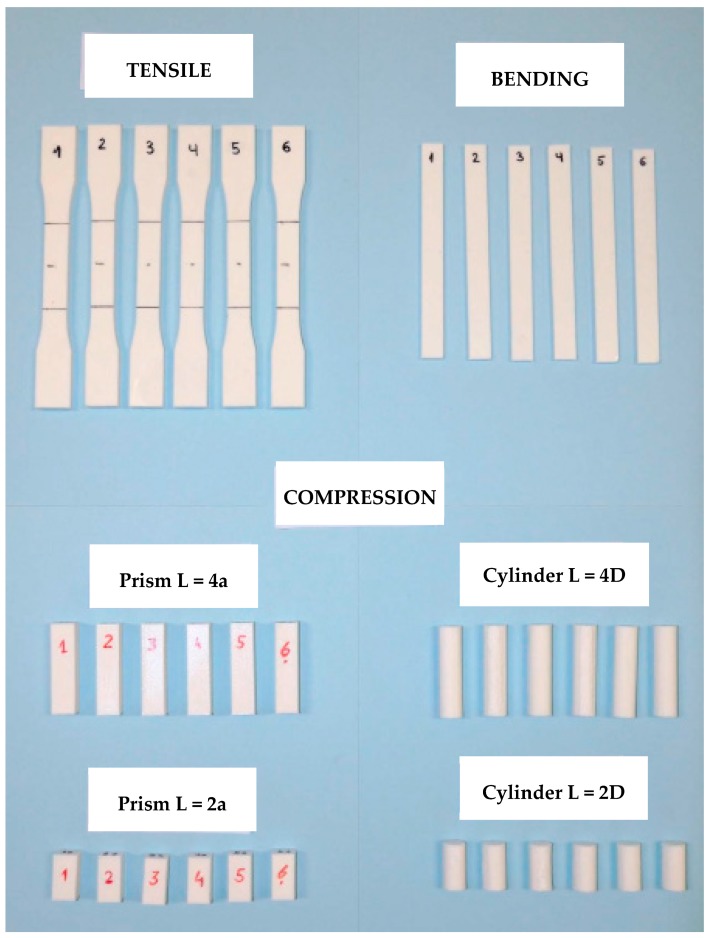
Poly Lactic Acid (PLA) samples for mechanical testing.

Figure 5 shows the 36 PLA 3D-printed samples grouped in six sets.

All test specimens were weighed on a KERN 400-55N precision scale (KERN & SOHN GmbH, Balingen, Germany), and measures were taken by means of a calliper to obtain the actual dimensions of each one of them. Tensile, compression and three-point bending tests were performed. All of them were carried out by means of an INSTRON machine model 3366 (INSTRON^®^, MA, USA) with the necessary equipment for each test.

### 2.2. Experimental Tests

#### 2.2.1. Tensile Test

**Figure 6:**
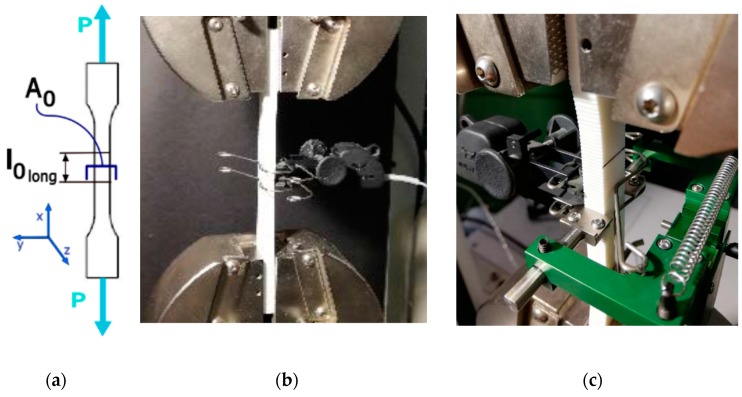
Tensile test assembly. Tensile test diagram (**a**); the longitudinal (**b**) and transverse (**c**) extensometers fitted on the specimen are shown.

According to the ASTM D638 standard [29], the speed of testing was set at 5 mm/min and the load–extension curve of the specimen was recorded. Longitudinal (INSTRON 2630-102) and transverse (INSTRON I3575-250M-ST) strain measuring devices (extensometers) were attached to the specimen in order to determine the Poisson’s ratio (Figure 6) (INSTRON^®^, MA, USA).

From the data recorded during the test, the values of stress (*σ*), strain (*ε*) and modulus of elasticity (*E*) were calculated (Equation (1)).

(1) $$\begin{array}{ccc} \left\{\sigma =\frac{P}{A_{0}}\right. & \varepsilon =\frac{\Rightarrow l}{l_{0}} & E=\frac{\sigma }{\varepsilon } \end{array},$$

where:

*P* = tensile load;

*A_0_* = initial cross-sectional area;

Δ*l* = increment of distance between gauge marks;

*l_0_* = initial specimen gauge length.

#### 2.2.2. Compression Test

**Figure 7:**
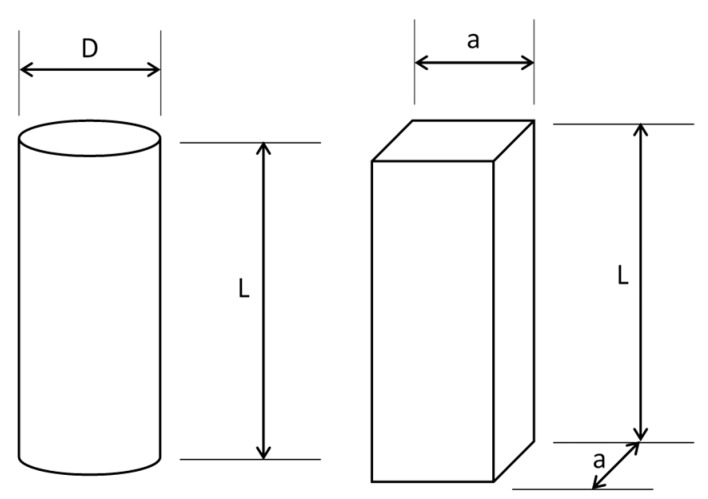
Dimensions of compression test specimens.

In the case of the compression test, the ASTM D695 standard [30] specifies that the test specimen shall be in the form of a right cylinder or prism (square), whose length is twice its width or diameter. However, when the modulus of elasticity and offset yield-stress are desired, the test specimen shall be of such dimensions that the slenderness ratio (λ) is in the range from 11 to 16:1. In this case, the preferred specimen sizes were 12.7 (a) by 12.7 (a) by 50.8 mm (L) (prism), or 12.7 in diameter (*D*) by 50.8 mm (L) (cylinder) (Figure 7). In literature consulted concerning this test, the length of the specimens was usually twice its principal width or diameter. Exceptionally [32,33,34,35] long-length specimens were tested.

(2) $$\lambda =\frac{L}{i}=\frac{L}{\sqrt{\frac{I}{A}}}=\left\{\begin{array}{l} \frac{L}{\sqrt{\frac{\frac{\pi }{64}D^{4}}{\frac{\pi }{4}D^{2}}}}=\frac{4L}{D}\in \left[11,\;16\right]\Rightarrow L\in \left[2.75D,\;4D\right]\;\;\;\mathrm{for}\;\mathrm{cylinder}\\ \frac{L}{\sqrt{\frac{\frac{1}{12}a^{4}}{a^{2}}}}=\frac{2\sqrt{3}L}{a}\in \left[11,\;16\right]\Rightarrow L\in \left[3.2a,\;4.6a\right]\;\;\;\mathrm{for}\;\mathrm{prism}\;\left(\mathrm{square}\right) \end{array}\right.,$$

where:

*i* = least radius of gyration;

*I* = moment of inertia;

*A* = area of the cross-section.

Limiting the slenderness ratio (λ) to the range 11 to 16:1 avoids 1) the influence of the end conditions on the results and 2) the buckling of the sample in the elastic range of the test.

Four series of specimens were built, called short specimens (L = 2a, L = 2D) and long specimens (L = 4a, L = 4D), whose length values were in the range given by Equation (2).

The speed of testing was set at 1.3 mm/min for short specimens (L = 2a, L = 2D) in accordance with paragraph 9 of the standard, and at 2.6 mm/min for long specimens (L = 4a, L = 4D), thus preserving the constant strain rate in all tests.

**Figure 8:**
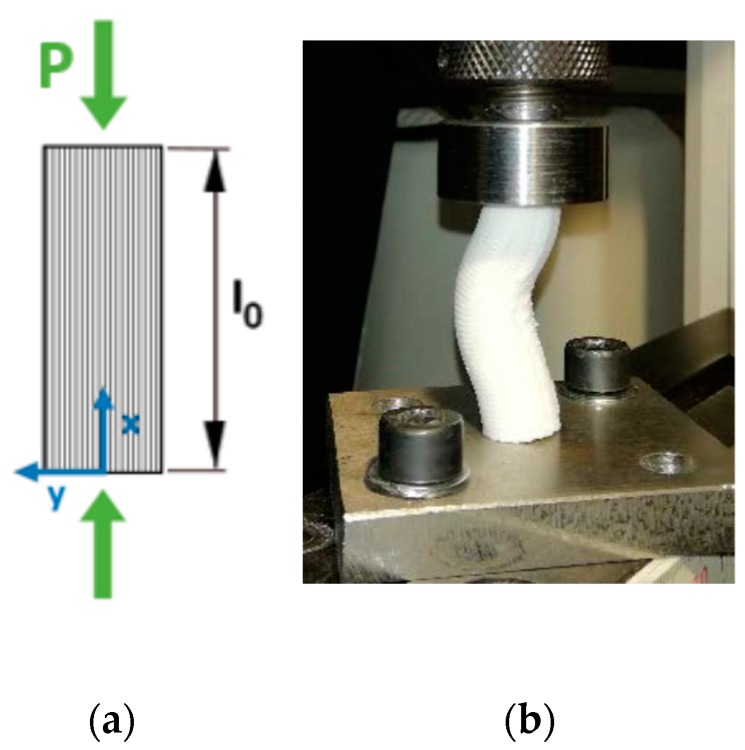
Compression test diagram (**a**); long specimen at the end of the test (**b**).

The test setup is shown in Figure 8.

In a similar way to the tensile test, the values of stress (σ), strain (ε) and modulus of elasticity (E) were calculated (Equation (1)).

#### 2.2.3. Three-Point Bending Test

**Figure 9:**
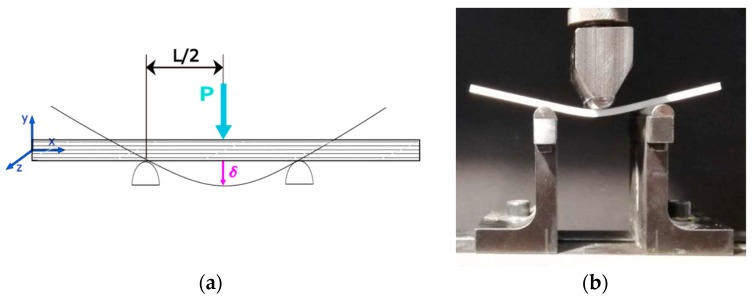
Bending test diagram (**a**); specimen under loading (**b**).

The three-point bending test was performed in accordance with ASTM D790 [31]. Figure 9 illustrates the test setup. The dimensions of the specimens can be seen in Figure 3.

The machine was set for the rate of crosshead motion (*R*) calculated according to Equation (3) taken from the standard:

(3) $$R=\frac{ZL^{2}}{6h}=\frac{0.01\times 52^{2}}{6\times 3.2}\approx 1.4\;mm/min,$$

where:

*L* = support span (52 mm);

*h* = depth of beam (3.2 mm);

*Z* = rate of strain of the outer fibre (mm/mm/min), where Z shall be equal to 0.01.

The test ended when the deformation reached 5%, which was equivalent to a vertical displacement (*δ*) of 7 mm (Procedure A).

The flexural stress (*σ_f_*) and flexural strain (*ε_f_*) were calculated according to Equation (4). The flexural modulus of elasticity (E_B_) is calculated from Equation (5).

(4) $$\left\{\sigma_{f}\begin{array}{cc} =\frac{3PL}{2bh^{2}} & \varepsilon_{f}=\frac{6\delta h}{L^{2}} \end{array}\right.,$$

where:

*P* = load at a given point on the load–deflection curve (N);

*b* = width of beam tested (mm)

### 2.3. Numerical Test

Finite Element (FE) analyses were carried out previously to reproduce mechanical testing on ABS 3D-printed parts [36,37]. When using experimental data in numerical models, it is important to ascertain under what conditions the mechanical properties were obtained [34].

This section presents a first approximation of a finite element model of the compression test. A three-dimensional model is shown that does not include the internal structure of the material. The software used was ANSYS^®^ Academic Research Mechanical, Release 19.1 [38].

**Figure 10:**
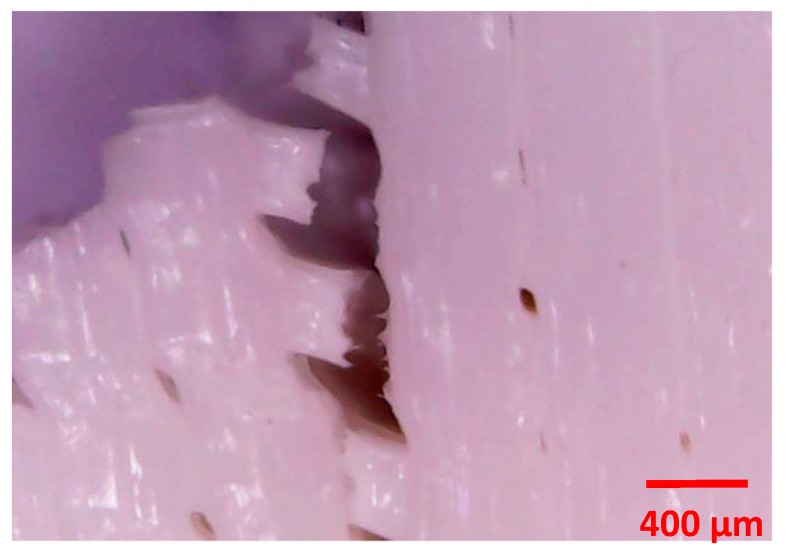
Enlargement of the surface area of a sample, where the porous nature of the material can be seen.

**Figure 11:**
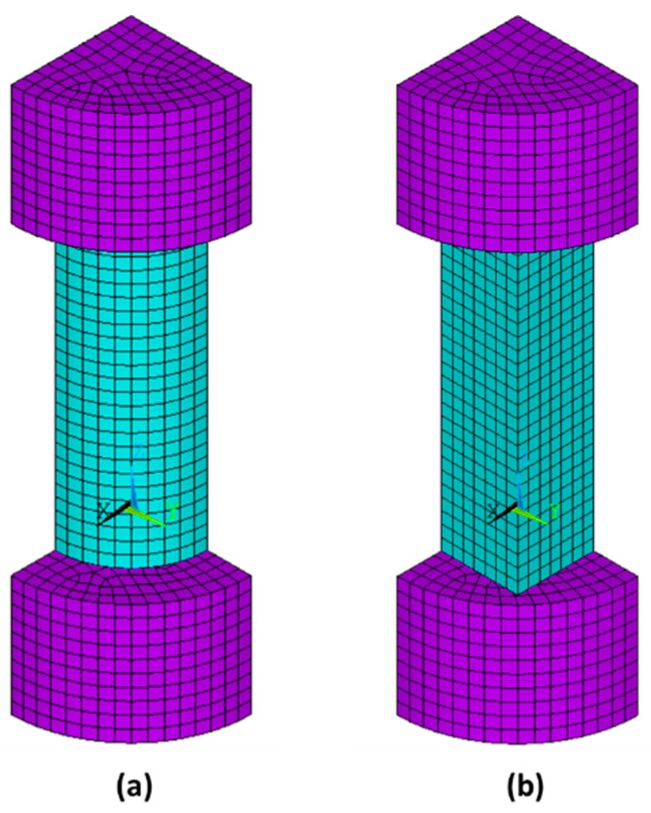
Finite element model for the cylindrical (**a**) and prismatic (**b**) short specimens. The purple elements correspond to the steel plates of the testing equipment.

- The simplified hypotheses assumed in the model were:
- Nominal dimensions were used to define the geometry of the sample
- The behaviour of the material was assumed to be linear, elastic and isotropic
- Neither the porosity of the material nor the manufacturing process was simulated. Figure 10 shows an enlarged image of the surface of one of the specimens, where the porosity and internal structure of the material is seen.
- Only a quarter of the specimen was modelled due to the symmetry of the analysis (Figure 11).

The features of the analysis were:

- Finite element Solid 186—3D, 20-node—was used for prismatic and cylindrical specimens;
- Material data (E,ν) found from experimental tests were introduced;
- The analysis was geometrically non-linear (GNA). Through an iterative process (Newton–Raphson), an equilibrium of forces was reached at each load step. The loading process was controlled by displacements.

The analysis reproduced the conditions of the compression test. The steel plates of the test setup were also simulated, and a contact between the specimen and the steel was introduced with a coefficient of friction of approximately 0.4 [39]. To define the boundary conditions, the bottom line of the bottom steel plate was fixed with zero displacement in all directions, and a negative vertical displacement was defined in the top steel plate. Symmetry boundary conditions were introduced at the two planes of symmetry.

## 3. Results

### 3.1. Results of Experimental Tests

#### 3.1.1. Tensile Test Results

**Figure 12:**
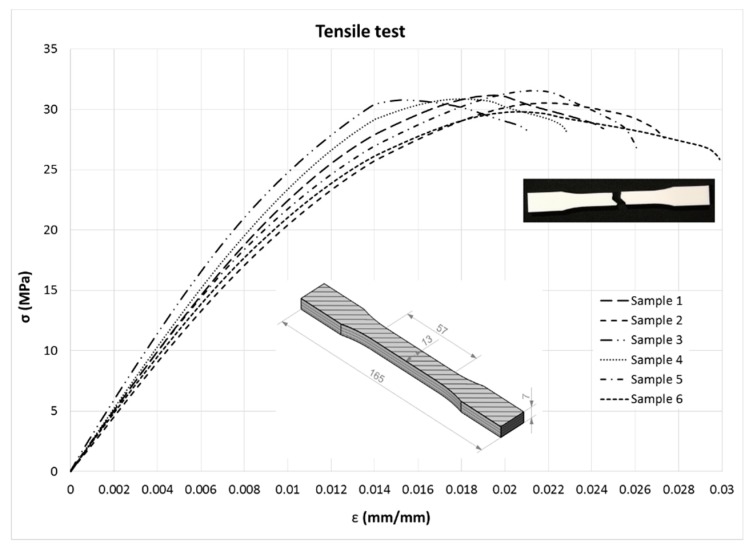
Tensile stress–strain curves.

**Figure 13:**
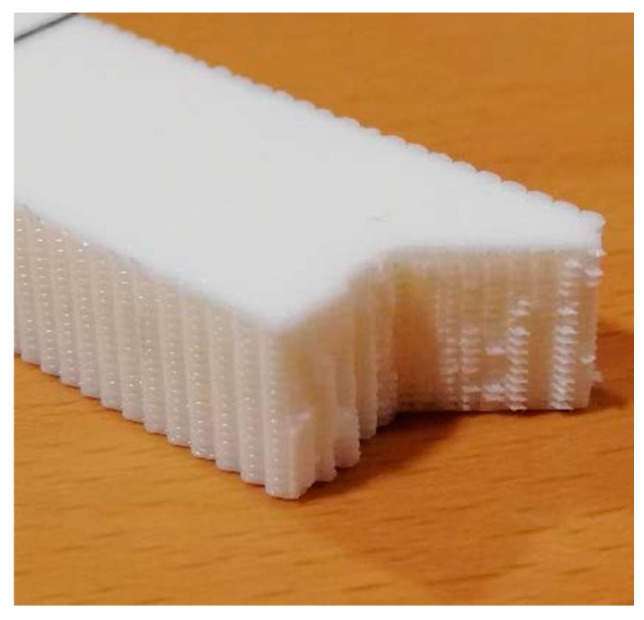
Aspect of brittle break of a tensile specimen by the minimum cross-section—7 ×19 (mm^2^).

Figure 12 shows the stress–strain curves, overlapped for all specimens. Figure 13 illustrates the tensile failure of a sample after the test.

The modulus of elasticity (E), strength (σ), elongation (ε) and Poisson’s ratio (ν) were derived from the data recorded during the test and subsequent calculations following the recommendations of the standard (Equation (1)).

**Figure 14:**
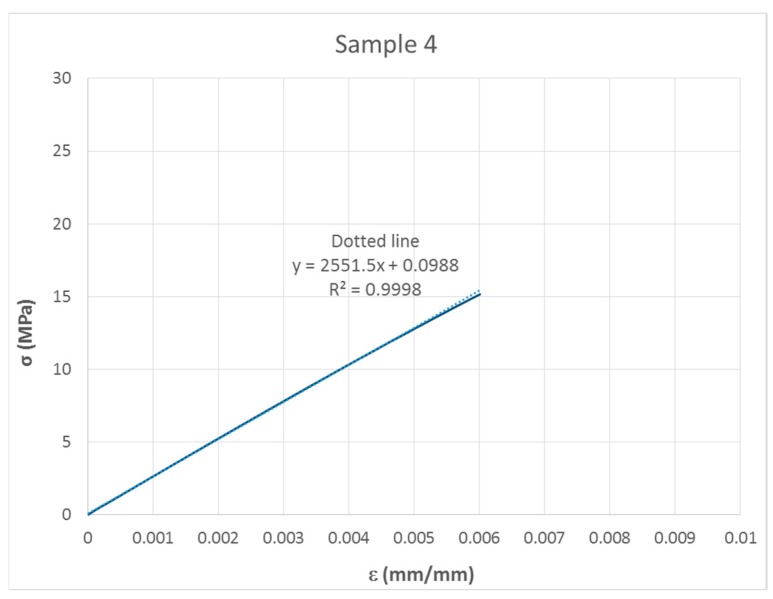
Regression line (dotted line) taken for calculation of modulus of elasticity (E).

The regression line of the initial linear part of the stress–strain curve was plotted, and the slope was taken as an E value (Figure 14).

**Figure 15:**
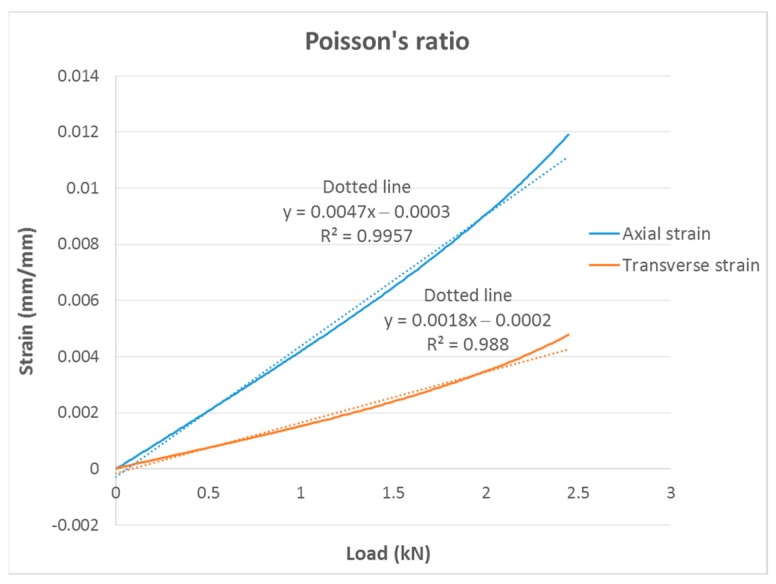
Regression lines (dotted lines) taken to calculate the Poisson’s ratio. The continuous lines represent the strain measured by the extensometers as a function of the applied load.

The Poisson’s ratio (ν) was calculated from the data recorded by the two extensometers (linear and transverse). Figure 15 shows both deformations as a function of the applied load. With the slopes of these two straight lines, ν was calculated by dividing the values by each other.

Values of modulus of elasticity (E), tensile strength (σ), elongation at tensile strength (ε) and Poisson’s ratio (ν) derived from tensile tests are shown in Table 3, including mean and standard deviation (SD).

#### 3.1.2. Compression Test Results

Analogously to the methodology followed for the tensile tests, from the force-displacement data recorded during the test, the stress–strain curve was obtained. The graphs were adjusted by doing the toe compensation indicated by the Annex A1 of the ASTM D 695 standard [30]. It consisted of ignoring the initial “toe” region of the stress–strain curve, and obtaining the corrected zero point of the stress axis by intersecting the prolongation of the linear region of the curve with the stress axis.

The modulus of elasticity (E), strength (σ) and elongation (ε) were determined from the data recorded during the test and subsequent calculations (Equation (1)).

It can be seen that, apparently, the strength increased in the plastic range of the material. This was because, as a result of compression, transformations occurred in the material, porosity was reduced, volume decreased and density increased. When the internal structure of the material changed, the load capacity changed. However, this did not affect the calculated parameters.

Values of modulus of elasticity (E), compressive yield point strength (σ) and elongation at compressive yield point strength (ε) are shown in Table 4, Table 5 and Table 6, including mean and standard deviation (SD). Since there were four series of test specimens in this case, it was deemed convenient to show the results separately (i.e., a table for each magnitude).

**Figure 16:**
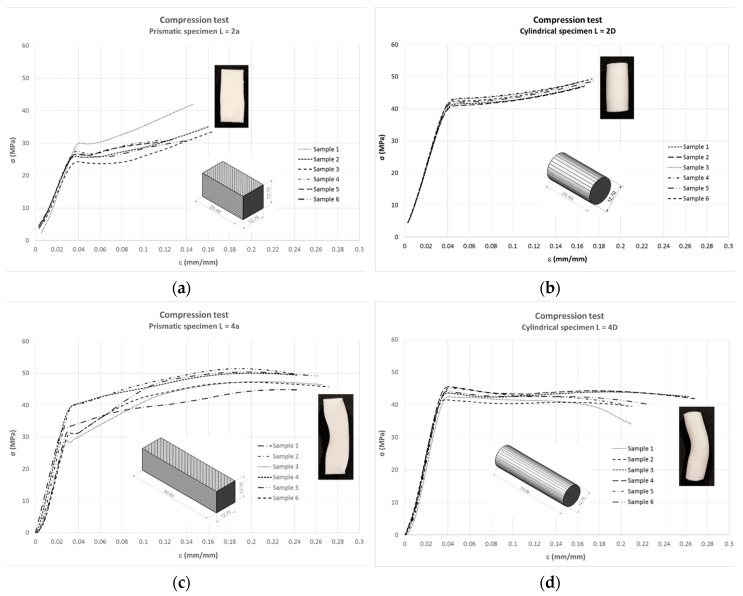
Compressive stress–strain diagrams: prismatic short shape (**a**), cylindrical short shape (**b**), prismatic long shape (**c**) and cylindrical long shape (**d**).

**Figure 17:**
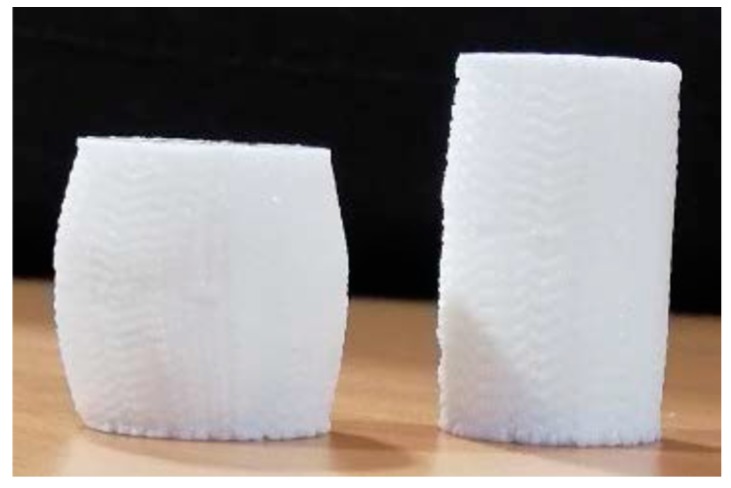
Samples tested until different levels of deformation.

Figure 16a,b shows the compressive response at medium levels of deformation for short specimens. For high levels of deformation, the long specimens failed by global buckling (Figure 16c,d), and the failure pattern of the short specimens was a barrel shape (Figure 17).

#### 3.1.3. Bending Test Results

**Figure 18:**
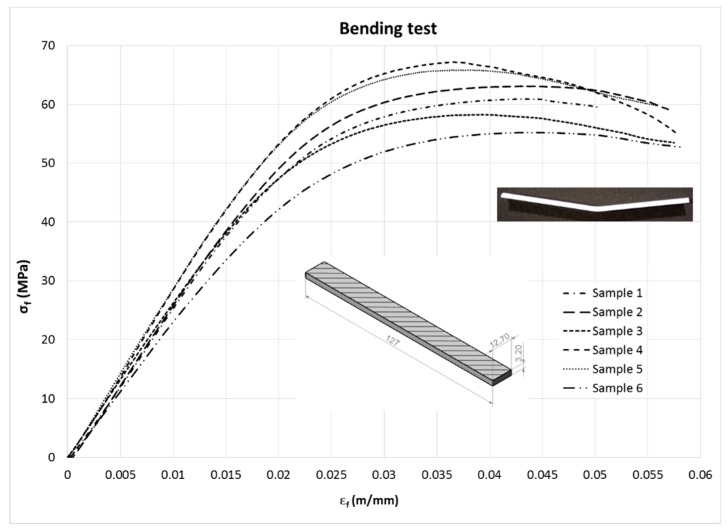
Flexural stress versus flexural strain curves.

Stress–strain curves, overlapped for all specimens, are shown in Figure 18. The maximum value of σ_f_ was taken as flexural strength, and ε_f_ was the respective deformation.

Values of the tangent modulus of elasticity (E_B_), flexural stress (σ_f_) and flexural strain (ε_f_) are shown in Table 7, including mean and standard deviation (SD). Flexural stress (σ_f_) and flexural strain (ε_f_) were calculated using Equation (4). According to the standard, the modulus of elasticity in bending (E_B_) was calculated by means of Equation (5):

(5) $$E_{B}=\frac{L^{3}\frac{P}{\delta }}{4bh^{3}}=\frac{PL^{3}}{48\delta I},$$

where:

*I* is the moment of inertia of the cross-section (mm^4^).

### 3.2. Numerical Testing Results (FE Analysis)

**Figure 19:**
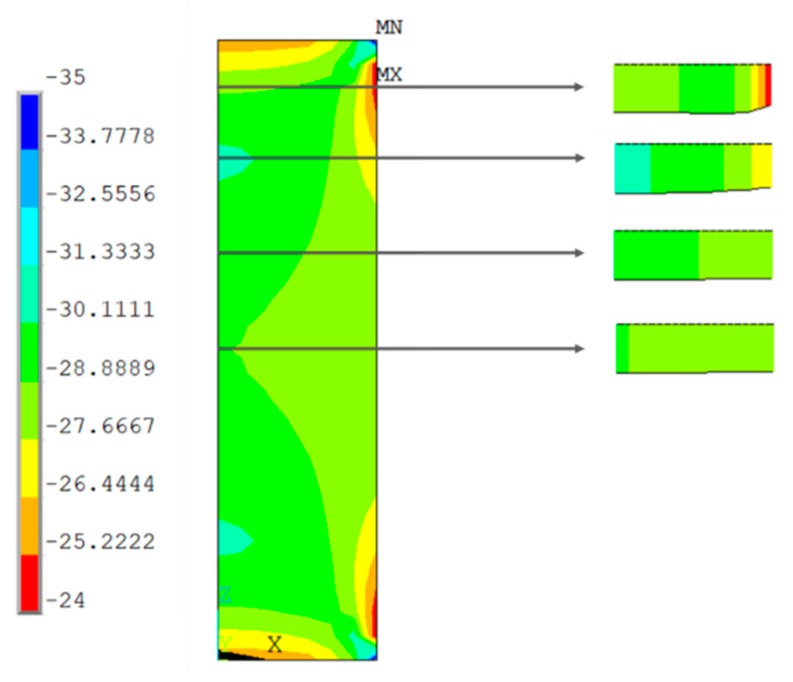
Distribution of normal compressive stresses (N/mm^2^) for the middle section of the symmetry plane. Short prismatic specimen.

**Figure 20:**
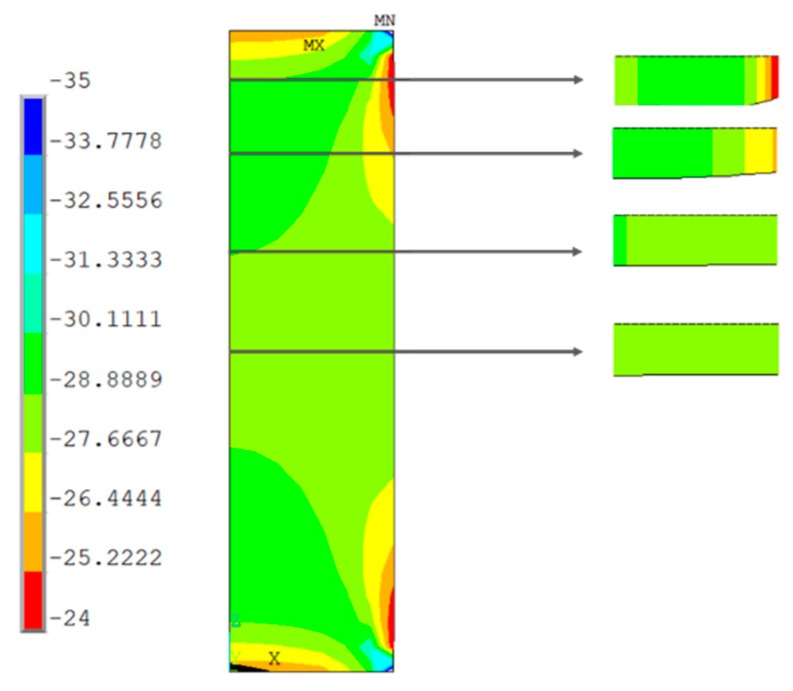
Distribution of normal compressive stresses (N/mm^2^) for the middle section of the symmetry plane. Short cylindrical specimen.

**Figure 21:**
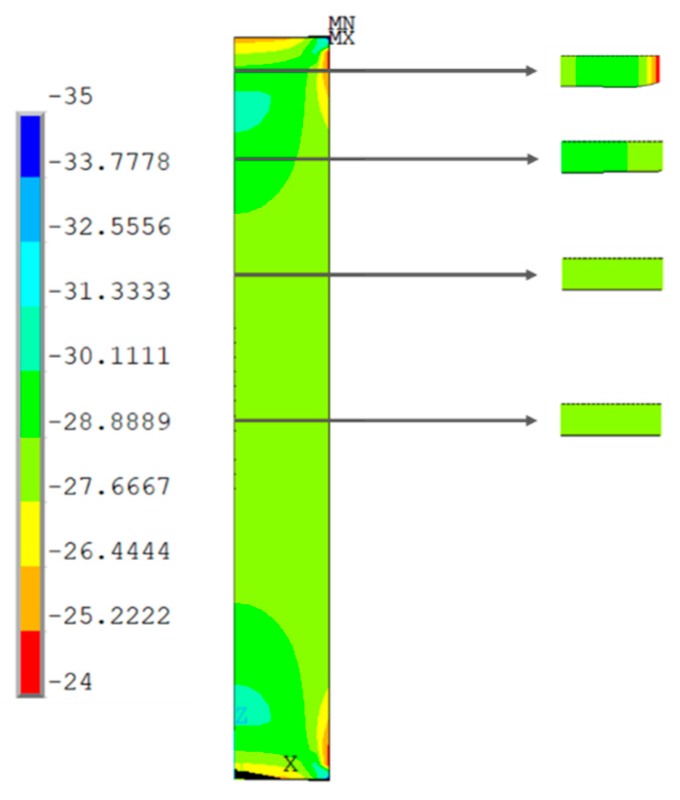
Distribution of normal compressive stresses (N/mm^2^) for the middle section of the symmetry plane. Long prismatic specimen.

**Figure 22:**
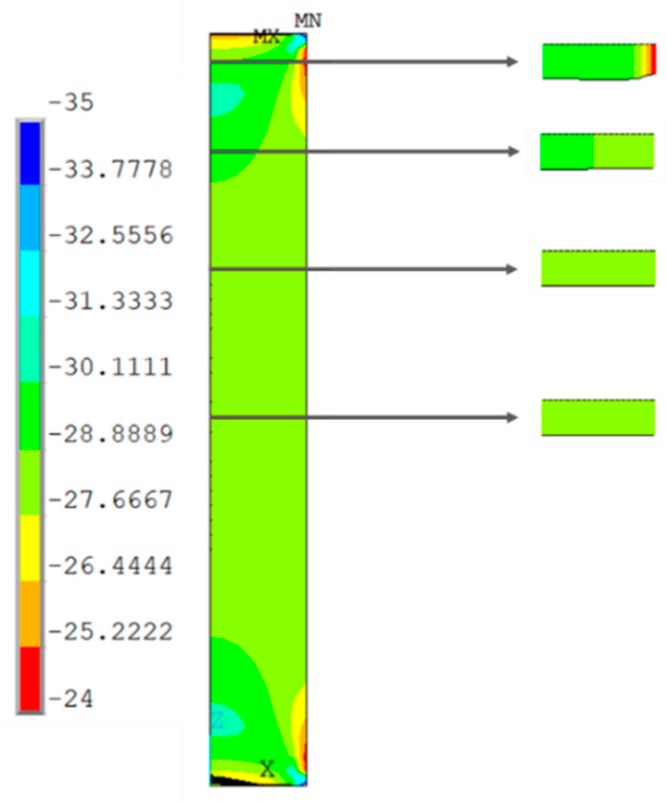
Distribution of normal compressive stresses (N/mm^2^) for the middle section of the symmetry plane. Long cylindrical specimen.

Figure 19, Figure 20, Figure 21 and Figure 22 show the normal stress plots for each of the four specimen types: short prismatic (Figure 19), short cylindrical (Figure 20), long prismatic (Figure 21) and long cylindrical (Figure 22).

There were no significant differences in the influence of the shape of the cross-section.

It can be perceived that longer samples exhibited a quasi-uniform distribution of stresses over a wide intermediate length, while shorter samples had hardly a uniform distribution in the intermediate cross-section, which means that the boundary conditions at the ends of the specimen had more influence on shorter samples. This effect was more notorious in the prismatic rather than the cylindrical specimens.

## 4. Discussion of Results

### 4.1. Experimental Tensile, Compression and Bending Tests

In order to compare the results between the three types of tests, in the case of the compression test, the average of the long specimens was taken, given that the standard recommends this when determining the modulus of elasticity.

**Figure 23:**
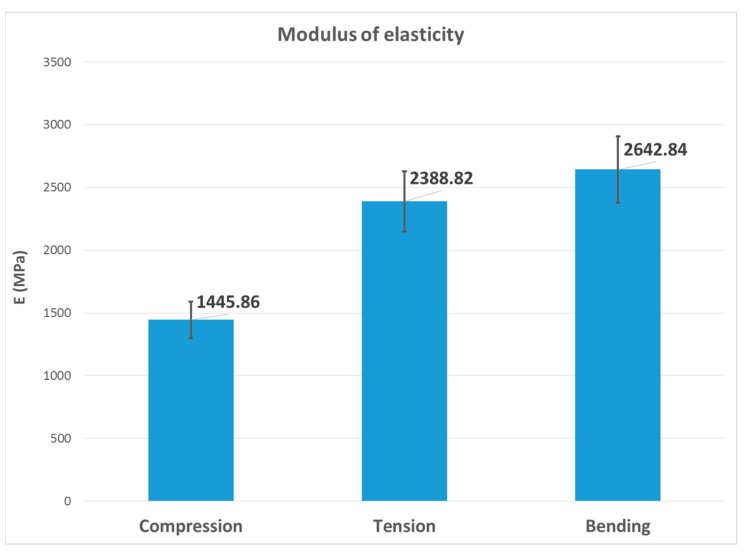
Bar graph with the mean value of the E variable.

**Figure 24:**
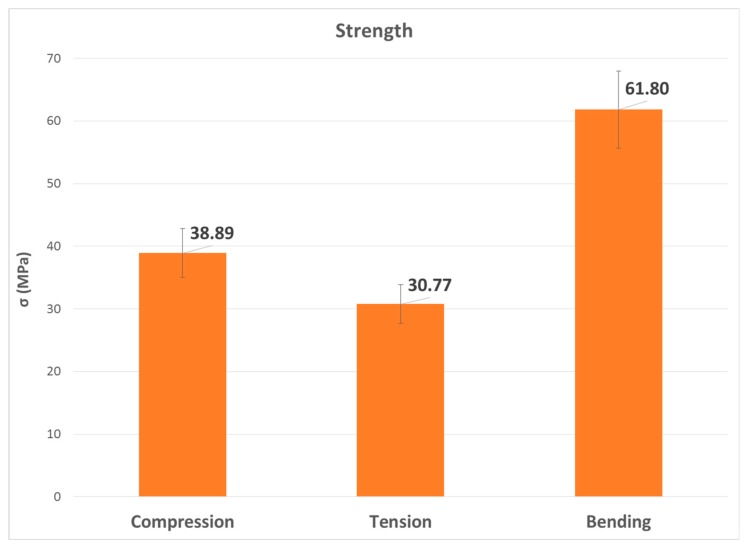
Bar graph with the mean value of the σ variable.

**Figure 25:**
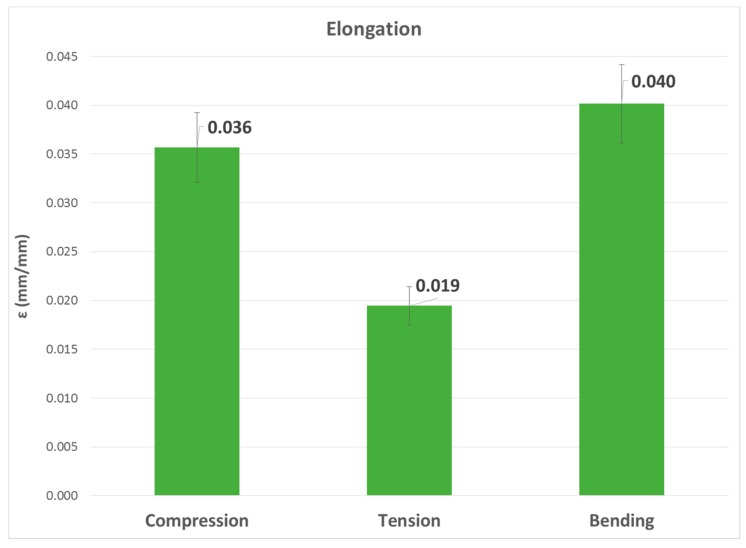
Bar graph with the mean value of the ε variable.

The second, third and fourth columns of Table 8 summarize the modulus of elasticity (E), strength (σ) and elongation (ε) found for each type of test, respectively, while the last two columns capture mechanical properties of processed PLA by injection [2,22]. Figure 23, Figure 24 and Figure 25 show the results in bar graph format.

It can be seen that the highest modulus of elasticity was the flexural modulus, with a value of 2640 MPa, followed by the tensile modulus with 2390 MPa. This was not a significant difference; however, the value of the modulus of elasticity in compression was 1445 MPa, approximately 40% lower than the previous two. This scenario would be anomalous from the point of view of a bimodular behaviour, as it should be an intermediate value between them. In effect, using the calculation method described in [23], given the elastic modules of 2515 MPa in tension and 1445 MPa in compression, the modulus in three-point bending should be around 1836 MPa (the effect of shear deformation in the specimen was taken into account), considerably lower than the observed value of 2575 MPa.

This apparent anomaly was investigated by re-evaluating the values of the elastic modules in order to find out the causes of the discrepancy. The following corrections were made to the data sets of the three tests:

- The nominal definitions of stress (force per unit of initial area) and strain (increase in length per unit of the initial length of the extensometer) were changed to their true values (true stress—force per unit of area of the deformed section; true strain—logarithmic strain), assuming the constant volume hypothesis (the volume of the material remains constant during deformation), in order to consider the effect of the variation of the cross section during the tensile and compression tests.
- The non-linearity in the initial phase of the stress–strain curve in the compression test was corrected for the long samples (L = 4D and L = 4a) by performing the toe compensation indicated in annex A1 of standard D695-02a. The parallelism between the faces of the samples was critical. This effect was more pronounced in short samples than in long samples.
- Calculations of the initial elastic modulus were carried out for the three types of tests (tension, compression and bending) considering the linear part of the stress–strain curves (strain from 0 to 0.004 mm/mm).

Table 9 lists the new elastic modulus values found after these adjustments.

The new values obtained showed less dispersion, both between samples of the same test and between the different types of cross-sections in the compression tests of long specimens.

As can be seen, the values of the tensile and bending modules were now more similar to those shown in Figure 23, although the mean bending value was slightly higher than the mean tensile value.

However, the results obtained from the three standard tests were still inconsistent with bimodular behaviour. This inconsistency is mainly attributed to differences in sample geometry, which also generates process differences, and to different strain rates in the standard tests. Since these are uniaxial tests, and the print layers are very thin, the effect of anisotropy was considered to be minor.

The most remarkable thing about graphics related to strength is that the flexural value was considerably higher than the other two, 60% higher than the compressive strength and twice as high as the tensile strength, which is clearly the lowest.

Finally, the graphs of the elongation values were compared. As above, the highest value corresponded to the bending test, followed by compression, although they did not show such a significant difference. The elongation for the tensile test was the lowest.

These results are shown here in summary:

$$E_{bending}\approx E_{tensile}\gg E_{compression}$$

$$\sigma_{bending}\gg \sigma_{compression}>\sigma_{tensile}$$

$$\varepsilon_{bending}≳\varepsilon_{compression}\gg \varepsilon_{tensile}$$

The results obtained from the tests of the four series of compression specimens, two lengths and two cross-section shapes, are analysed in the following Section.

### 4.2. Specific Analysis of the Compression Test

**Figure 26:**
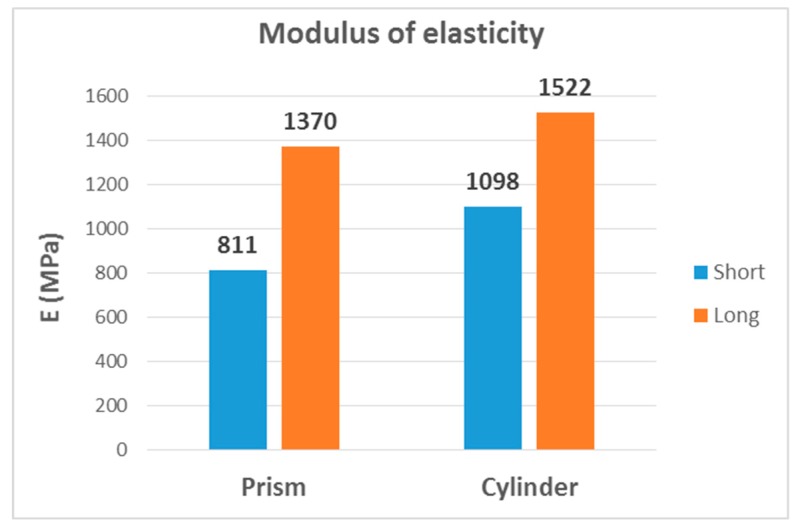
Comparison of modulus of elasticity in compression tests.

As can be seen in Figure 26, the modulus of elasticity of long specimens was considerably higher regardless of the cross-section shape. This result is consistent with [34]: Young’s modulus decreases when the diameter:length ratio increases. In the case of cylindrical specimens, the value for long specimens was 40% higher, being 70% higher in prismatic specimens. On the other hand, the cylinder shape also had a higher value within each length. Thus, the highest value was that of long-cylinder specimens.

In the last row of Table 4, the dispersions of the values of the compressive modulus of elasticity (in %) were calculated for each type of sample. All values are within the acceptable range of variation when the modulus of elasticity is measured, as this is a parameter that tends to present higher dispersion than other ones (e.g., breaking stresses). Some conclusions can be drawn from Table 4:

(a) It was observed that the rectangular samples (2a and 4a) showed higher dispersion (in %) than the cylindrical ones (2D and 4D). Therefore, it is recommended the use of cylindrical specimens to determine the modulus E.
(b) The difference between the mean value of the modulus of elasticity obtained from samples 4D and 4a was 11.0 %, whereas the difference between the mean value obtained from the 2D and 2a samples was 35.3%.

By combining reasoning a) and b), it is concluded that the most appropriate sample type to determine the modulus E is the L = 4D type.

In addition to the dispersion inherent in a compression test, the influence of two other factors was identified and analysed:

(c) The flexibility of the testing machine caused an increase in the displacement between the compression plates, which implied obtaining an elastic modulus lower than the real modulus of the material. Although the compression test standard ASTM D695-02a did not provide for correction for this effect, the actual modulus E can be calculated from the measured modulus E’ by using the following Equation:
  (6) $$E=\frac{L}{\frac{L}{E^{\prime }}-\frac{A}{K_{M}}},$$

where:

*L* is the length of the specimen;

*A* is the area of the cross-section;

*K_M_* is the rigidity of the testing machine.

Table 10 shows the values initially determined for each type of specimen together with the values achieved after making the correction for the flexibility of the testing machine, as well as the variation (in %).

It was observed that the effect of the flexibility of the testing machine was less for long specimens (4a and 4D). This is an additional reason for using long specimens to determine the modulus E.

(d) Friction between the sample and the load plates of the machine limited the effect of Poisson at the ends of the sample, and caused a barrel shape, which altered the assumed uniform distribution of stresses and deformations, thus increasing the apparent elastic modulus. The barrel shape was observed in some of the specimens (see Figure 17). To verify the influence of this effect on the Young’s modulus, two finite element simulations of the compression tests were performed. In the first one, it was assumed that there was no friction, and in the second one, the friction completely blocked the sliding between both surfaces in contact. The sample stiffness variation (which is proportional to the Young’s modulus) is shown in Table 11, where the percentage compression stiffness variation is expressed with regard to the frictionless model.

It was noticed that the influence of the coefficient of friction between the specimen and the test machine plates was low, being lower for long specimens (4a and 4D). This is an additional argument for using long specimens to determine the modulus E.

**Figure 27:**
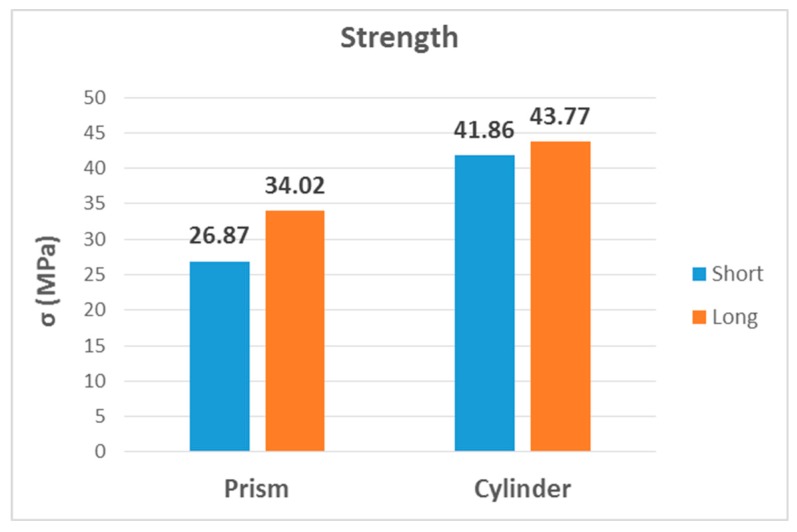
Comparison of compressive strength.

In the values of compressive strength (Figure 27), it is observed that cylindrical samples had higher strength values, regardless of the length of the sample. The differences between them were not as noticeable as in the case of the modulus of elasticity.

**Figure 28:**
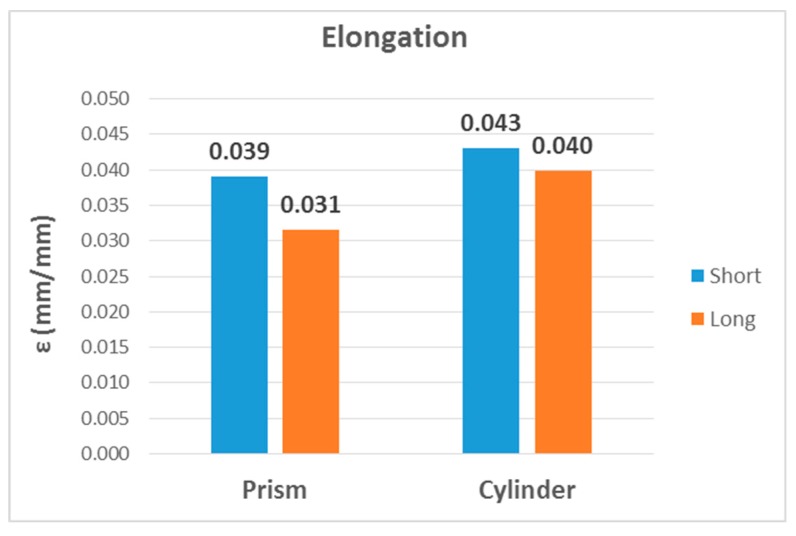
Comparison of elongation in compression tests.

Finally, the elongation (Figure 28) obtained for cylindrical samples was also slightly higher than that obtained for prismatic samples. In addition, higher values were found in the short samples than in the long ones. However, in the case of elongation, the differences were less meaningful than in the case of the modulus of elasticity and strength.

These results are summarized in the following expressions:

$$E_{Cylinder}>E_{Prism}\;and\;E_{Long}\gg E_{Short}$$

$$\sigma_{Cylinder}>\sigma_{Prism}\;and\;\sigma_{Long}\approx \sigma_{Short}$$

$$\varepsilon_{Cylinder}>\varepsilon_{Prism}\;and\;\varepsilon_{Long}<\varepsilon_{Short}$$

## 5. Conclusions

The mechanical properties of PLA manufactured by FDM were determined under tensile, compressive and flexural stresses. The results obtained are quite consistent, considering the low dispersion of results within each group of specimens and in comparison with available data.

However, the results obtained in this work show that PLA has a double asymmetry in its tensile and compressive behaviours: On the one hand the asymmetry in strength, and on the other hand asymmetry in the constitutive behaviour, which suggests the need to treat this material by means of a bimodular elasticity model.

Nevertheless, characterisation based on standard tests presents significant difficulties when its purpose goes beyond its application to quality control tasks, such as numerical simulation.

The behaviour of 3D-printed materials is highly sensitive to process factors and, in the case of polymeric materials, also to the effect of different strain rates applied in each test. This makes it difficult to achieve a consistent characterisation of the elastic constants among the various types of standard tests available today, even when taking into account that the different dimensions and shapes of the specimens in each test can cause process differences that affect in the measured properties.

For this reason, it is necessary to define a new characterisation procedure that allows obtaining a consistent set of elastic constants, with the minimum number of tests possible, especially adapted to bimodular materials. This result would be of great interest for carrying out simulations of the structural behaviour of 3D printed parts. Recently, Mazzanti et al. [40] concluded there is no recognised international standard governing the characterisation of the tensile, compressive or flexural properties of 3D-printed materials. The current standards are those used for the characterisation of bulk polymeric materials. In this case, the geometric characteristics are standardized through the concepts of stress and strain, but in the case of 3D printing, this is difficult because the specimen is actually a structure, not a material.

With respect to the compression test, it has been demonstrated that there is less variability of results in cylindrical specimens than in prismatic specimens (probably a result of the manufacturing process, since at the corners of the prismatic shapes the printing head can deposit excess material). In addition, when determining the modulus of elasticity, it is confirmed that, following the recommendation of the ASTM D695 standard, the longer specimens provide results that are more consistent.

The need to perform compression tests to characterise the elastic modulus to compression should be reconsidered. This could be consistently deduced using a bimodular model from the bending test, through the flexural modulus, or also from the shear test, through the transverse modulus of elasticity G, thus avoiding the uncertainties associated with the compression test.

The simulation of the compression test shows that the model is sensitive to the boundary conditions applied at the ends of the specimen (friction, etc.). Despite the introduction of geometric non-linearity in the analysis (GNA), this is not enough to correctly reproduce the actual behaviour of the material in a coherent way.

## Figures and Tables

**Table 1:** Filament specifications and properties

Specification/Parameter	Value
Diameter (mm)	2.85
Specific gravity (ISO 1183) (g/cm^3^)	1.24
Tensile strength at yield (ISO 527) (MPa)	70
Strain at yield (ISO 527) (%)	5
Strain at break (ISO 527) (%)	20
Tensile modulus (ISO 527) (MPa)	3120
Melting temperature (ISO 11357) (°C)	115 ± 35
Glass transition temperature (ISO 11357) (°C)	57
Molecular weight (g/mol)	1.598 × 10^5^

**Table 2:** Printing parameters.

Parameter	Value
Infill ratio (%)	90
Nozzle diameter (mm)	0.4
Printing temperature (°C)	205
Printing speed (mm/s)	50
Printing pattern	Rectangular
Raster angle (°)	45
Layer height (mm)	0.1

**Table 3:** Tensile properties obtained from tensile tests (ASTM D638).

Specimen	Modulus of Elasticity E (MPa)	Tensile Strength σ (MPa)	Elongation at σ ε (mm/mm)	Poisson’s Ratio ν
1	2200.7	31.13	0.019	-
2	2063.6	30.52	0.022	-
3	2776.4	30.78	0.015	-
4	2551.5	30.84	0.018	0.38
5	2394.0	31.55	0.021	0.37
6	2346.7	29.82	0.021	0.36
Mean	2388.8	30.77	0.019	-
SD	252.8	0.59	0.002	-

**Table 4:** Modulus of elasticity from compression test (ASTM D695).

Specimen	E (MPa)			
	Prism (L = 2a)	Cylinder (L = 2D)	Prism (L = 4a)	Cylinder (L = 4D)
1	921.2	1076.1	1397.7	1472.4
2	799.6	1077.6	1458.4	1463.8
3	724.2	1099.2	1251.7	1516.2
4	816.4	1113.5	1453.0	1563.0
5	802.7	1097.6	1355.3	1564.5
6	802.4	1123.3	1304.9	1549.4
Mean	811.1	1097.9	1370.2	1521.5
SD	63.2	18.9	82.4	45.0

**Table 5:** Compressive yield point strength from compression test (ASTM D695).

Specimen	σ (MPa)			
	Prism (L = 2a)	Cylinder (L = 2D)	Prism (L = 4a)	Cylinder (L = 4D)
1	30.06	41.17	32.57	42.49
2	25.97	40.66	39.79	41.56
3	24.25	42.58	28.83	43.64
4	27.57	42.82	40.10	45.52
5	26.58	41.78	31.71	45.14
6	26.79	42.13	31.10	44.27
Mean	26.87	41.86	34.02	43.77
SD	1.92	0.83	4.76	1.53

**Table 6:** Elongation at compressive yield point strength from compression test (ASTM D695).

Specimen	ε (mm/mm)			
	Prism (L = 2a)	Cylinder (L = 2D)	Prism (L = 4a)	Cylinder (L = 4D)
1	0.041	0.043	0.026	0.042
2	0.039	0.043	0.035	0.038
3	0.039	0.044	0.029	0.041
4	0.038	0.043	0.036	0.042
5	0.038	0.043	0.031	0.043
6	0.039	0.042	0.032	0.033
Mean	0.039	0.043	0.031	0.040
SD	0.001	0.000	0.004	0.004

**Table 7:** Flexural properties from three-point bending test (ASTM D790).

Specimen	E_B_ (MPa)	σ_f_ (MPa)	ε_f_ (mm/mm)
1	2603.0	60.92	0.042
2	2657.4	63.19	0.043
3	2572.1	58.34	0.039
4	2900.2	67.26	0.036
5	2859.0	65.87	0.036
6	2265.4	55.22	0.043
Mean	2642.8	61.80	0.040
SD	229.0	4.57	0.003

**Table 8:** Summary table of the mechanical properties of the three tests.

Test	E (MPa)	σ (MPa)	ε (mm/mm)	Farah et al. [2]	*Song et al. [22]
Compression	1445.9	38.89	0.036	-	3200 ÷ 3880, 70.80 ÷ 71.94, −
Tensile	2388.8	30.77	0.019	3700, 65.6, 0.04	3430 ÷ 3680, 29.79 ÷ 46.76, −
Flexural	2642.8	61.80	0.040	-	-

(*) depending on the strain rate.

**Table 9:** Summary of re-evaluated modulus of elasticity.

Specimen	E (MPa)			
	Tension	Compression L = 4D	Compression L = 4a	Bending
1	2497	1405	1405	2455
2	2282	1362	1375	2666
3	2845	1485	1225	2679
4	2599	1507	1441	2699
5	2478	1452	1353	2601
6	2388	1459	1276	2349
Mean	2515	1445	1346	2575
SD	194	53	81	142

**Table 10:** Influence of the flexibility of the testing machine.

Specimen	E’ (Measured) (MPa)	E (Real) (MPa)	Variation (%)
L = 2a	811.1	938	16
L = 2D	1097.9	1283	17
L = 4a	1370.2	1547	13
L = 4D	1521.5	1690	11

**Table 11:** Variation of the stiffness with respect to the model without friction, obtained by FEA.

Specimen	Variation in Stiffness (%)
L = 2a	4.17
L = 2D	3.56
L = 4a	2.08
L = 4D	1.78
